# Long-Term Use of Tamoxifen Is Associated With a Decreased Subsequent Meningioma Risk in Patients With Breast Cancer: A Nationwide Population-Based Cohort Study

**DOI:** 10.3389/fphar.2019.00674

**Published:** 2019-06-12

**Authors:** Li-Min Sun, Cheng-Li Lin, Sean Sun, Chung Y. Hsu, Zonyin Shae, Chia-Hung Kao

**Affiliations:** ^1^Department of Radiation Oncology, Zuoying Branch of Kaohsiung Armed Forces General Hospital, Kaohsiung, Taiwan; ^2^Management Office for Health Data, China Medical University Hospital, Taichung, Taiwan; ^3^College of Medicine, China Medical University, Taichung, Taiwan; ^4^Department of Cardiovascular Science, Midwestern University, Glendale, AZ, United States; ^5^Graduate Institute of Biomedical Sciences and School of Medicine, College of Medicine, China Medical University, Taichung, Taiwan; ^6^Department of Computer Science and Information Engineering, Asia University, Taichung, Taiwan; ^7^Department of Nuclear Medicine, China Medical University Hospital, Taichung, Taiwan; ^8^Department of Bioinformatics and Medical Engineering, Asia University, Taichung, Taiwan

**Keywords:** breast cancer, meningioma, tamoxifen, population-based study, cohort study

## Abstract

**Background:** Earlier studies have indicated a relatively higher risk of occurring meningioma among female breast cancer survivors and have suggested that tamoxifen might decrease this risk. The present study evaluated whether tamoxifen use in breast cancer patients can reduce the risk of meningioma.

**Methods:** We designed a population-based cohort study by using data from the National Health Insurance system of Taiwan to assess this issue. Between January 1, 2000, and December 31, 2008, women with breast cancer and of age ≥20 years were included. They were divided into two groups: those who had not received tamoxifen therapy and those who had. The Cox’s proportion hazard regression analysis was conducted to estimate the effects of tamoxifen treatment and the subsequent meningioma risk.

**Results:** We identified a total of 50,442 tamoxifen users and 30,929 non-tamoxifen users. Tamoxifen users had a borderline significantly lower overall risk of meningioma than non-tamoxifen users [adjusted hazard ratio (aHR) = 0.64, 95% confidence interval (95% CI) = 0.40–1.02]. A statistically significant difference was found in those patients with tamoxifen treatment duration longer than 1,500 days (aHR = 0.42, 95% CI = 0.19–0.91) or with cumulative dosage exceeding 26,320 mg (aHR = 0.44, 95% CI = 0.22–0.88). Furthermore, no statistically significant joint effect of aromatase inhibitors and tamoxifen on the occurrence of meningioma among breast cancer patients was seen.

**Conclusion:** Tamoxifen users had a non-significantly (36%) lower risk of developing meningioma than did tamoxifen non-users; however, our data indicated that tamoxifen therapy is associated with a reduced meningioma risk for Taiwanese breast cancer patients receiving long duration or high cumulative dosage treatment with tamoxifen.

## Introduction

Among women in low-, middle-, and high-income countries, breast cancer is the most frequently occurring cancer, with an estimated 2.09 million new cases diagnosed worldwide in 2018 International Agency for Research on Cancer (IARC) and World Health Organization (WHO). Similarly, in Taiwan, breast cancer has been the most prevalent cancer among women for two decades, during which the age-adjusted incidence rate has risen gradually from 55.88 per 100,000 people in 2002 to 89.21 per 100,000 people in 2012 (Chiang et al., 2016). In the near future, increasing numbers of long-term survivors of breast cancer can be expected because of factors such as effective cancer screening, improved diagnostic tools that enable early detection, improved adjuvant treatments, relatively favorable prognoses with slower progression than most other cancers, and the aging population. As a result, surveillance and follow-up care of such survivors have become pertinent topics, both for the control of the cancer and its treatment-related health conditions (Howard-Anderson et al., 2012).

Meningioma originates in the meningothelial cells of the arachnoid membrane and is the most prevalent primary intracranial neoplasm in adults. Between 2008 and 2012, in the United States, meningioma accounted for 36.4% of all primary tumors occurring in the brain and other organs of the central nervous system (Ostrom et al., 2015). The rate of meningioma incidence in women is nearly two times that in men, especially during the reproductive period of life (Claus et al., 2008; Wiemels et al., 2010; Ostrom et al., 2015). Breast cancer in women has been associated with meningioma (Helseth et al., 1989; Lieu et al., 2003; Rao et al., 2009; Milano and Grossman, 2017). Approximately 66% of all breast cancers (and even higher in older women) are hormone receptor–positive (Rakha et al., 2007; Morrison et al., 2012). Thus, for most patients, hormone therapy should be considered as an adjuvant to surgery. Evidence suggests that tamoxifen—among the most extensively administered selective estrogen receptor modulators in hormone receptor–positive breast cancer patients—increases disease-free and overall survival rates (Lin and Winer, 2008). Even though tamoxifen is generally well tolerated, with a fair adherence rate (Wigertz et al., 2012), the safety of tamoxifen treatment is of clinical concern (Perez, 2007). The occurrence of meningioma and tamoxifen use might plausibly be connected; hormonal factors have been proven to be involved in the development of meningioma (Hsu et al., 1997; Wahab and Al-Azzawi, 2003; Lee et al., 2006; Korhonen et al., 2010; Cea-Soriano et al., 2012), and approximately 70% of meningiomas show progesterone receptor expression whereas approximately 30% show estrogen receptor expression (Wahab and Al-Azzawi, 2003). An early study suggested that tamoxifen treatment may prevent the development of meningioma in breast cancer patients (Ji et al., 2016).

In the present retrospective cohort study, we used a population-based database derived from Taiwan’s National Health Insurance (NHI) system to investigate whether tamoxifen treatment can effectively protect breast cancer survivors against meningioma development and whether the duration or cumulative dosage of tamoxifen treatment affects this possible relationship.

## Methods

### Data Source

Taiwan provides medical insurance coverage to 99% of its 23.74 million residents; data regarding medical insurance recipients are stored in the NHI Research Database (NHIRD) (Database NHIR). The Registry for Catastrophic Illness Patient Database (RCIPD), a subset of the NHIRD, was used for this retrospective cohort study. In Taiwan, when patients apply for qualified catastrophic illness certificates, their illnesses must be confirmed by clinical physicians. The RCIPD has been discussed in detail in the literature (Peng et al., 2015; Huang et al., 2016). The diagnoses in the NHIRD are coded on the basis of the International Classification of Diseases, Ninth Revision, Clinical Modification (ICD-9-CM).

### Study Population

In the data for the period from January 1, 2000, to December 31, 2008, we identified women with breast cancer and of age ≥20 years (ICD-9-CM 174). The identified patients were divided into two groups: those who had not received tamoxifen therapy (i.e., the non-tamoxifen cohort or controls) and those who had (i.e., the tamoxifen cohort). The index date in the tamoxifen cohort was set as the day of first tamoxifen treatment. For each patient in the non-tamoxifen cohort, the index date was a randomly selected date with the same index year as that in the tamoxifen cohort. Patients younger than 20 years and those with a history of meningioma (ICD-9-CM 225.2) or other cancers (namely, ICD-9-CM 140-173 and 175-208) prior to the index date were excluded.

### Outcome and Comorbidities and Medication

From the index date onward, both cohorts were followed until withdrawal from the NHI program, meningioma occurrence, or December 31, 2011. Patients whose claims records revealed a diagnosis of alcohol-related illness (ICD-9-CM 291, 303, 305, 571.0, 571.1, 571.2, 571.3, 790.3, A215, and V11.3), chronic obstructive pulmonary disease (COPD) (ICD-9-CM: 491, 492, and 496), coronary artery disease (CAD) (ICD-9-CM 410–414), diabetes (ICD-9-CM 250), hypertension (ICD-9-CM 401–405), or stroke (ICD-9-CM 430–438) at the baseline were defined as having comorbidities. The medications included steroids, statins, and thiazide diuretics. In addition, treatment information for breast cancer patients who received surgical operations, aromatase inhibitors, chemotherapy, and radiotherapy was considered in our study.

### Statistical Analysis

The distributions of age, comorbidities, drug, and breast cancer treatment were calculated as frequency (relative frequency, %) or mean ± standard deviation. The differences in the data of the two cohorts were analyzed through Student *t* testing for continuous variables and chi-square testing for categorical variables. We used the Kaplan–Meier method to assess the cumulative incidence of meningioma in the tamoxifen and non-tamoxifen cohorts and estimated the differences between the cohorts through log-rank testing. In addition, the incidence density of meningioma per 10,000 person-years was computed for each cohort. Univariable and multivariable Cox proportional hazards models were employed to calculate the hazard ratios (HRs) and 95% confidence intervals (CIs) of meningioma in the tamoxifen cohort relative to the non-tamoxifen cohort. Given that during the study period, the patients may have taken tamoxifen irregularly, the calculations here may have underestimated the drug effect. To diminish this bias in estimating the meningioma risk, we employed Cox proportional hazard model with time-dependent exposure covariates. We evaluated the effects of tamoxifen use duration (≤365, 366–1,500, and >1,500 days) and cumulative dosage (≤4,280, 4,281–12,980, 12,981–26,320, and >26,320 mg) on the risk of meningioma in patients with breast cancer. Furthermore, we assessed the joint effects of aromatase inhibitor use and tamoxifen use. All data were analyzed using the SAS statistical package (v9.4; SAS Institute Inc., Cary, NC, USA). Any difference with two-tailed *P* < 0.05 was considered statistically significant.

## Results

**Table 1** presents a comparison of the baseline characteristics of the two cohorts. On average, patients in the tamoxifen cohort were younger than those in the non-tamoxifen cohort. The non-tamoxifen cohort had higher proportions of patients with CAD, stroke, hypertension, diabetes, statin use, and thiazide diuretics use. The tamoxifen cohort exhibited higher proportions of breast surgery, radiotherapy, aromatase inhibitor alone, and combined aromatase inhibitor and chemotherapy treatment; however, the non-tamoxifen cohort had a higher proportion of chemotherapy alone.

**Table 1:** Demographic and comorbidity data of breast cancer patients classified by tamoxifen use status.

	Breast cancer						*P* value
	All *n* = 81,371		Without tamoxifen use *n* = 30,929		With tamoxifen use *n* = 50,442		
**Variable**	***n***	**%**	***n***	**%**	***n***	**%**	
**Age, years**							<0.001
20–44	21,556	26.5	7,075	22.9	14,481	28.7	
45–64	47,179	58.0	18,588	60.1	28,591	56.7	
≥65	12,636	15.5	5,266	17.0	7,370	14.6	
Means (SD)^a^	52.6	11.7	53.7	11.9	51.9	11.6	<0.001
**Comorbidity**							
CAD	10,426	12.8	4,069	13.2	6,357	12.6	0.02
COPD	6,308	7.75	2,452	7.93	3,856	7.64	0.14
Stroke	1,901	2.34	891	2.88	1,010	2.00	<0.001
Alcohol-related illness	1,220	1.50	459	1.48	761	1.51	0.78
Hypertension	23,924	29.4	9,506	30.7	14,418	28.6	<0.001
Diabetes	8,092	9.94	3,272	10.6	4,820	9.56	<0.001
**Drug**							
Steroids	47,754	58.7	18,009	58.2	29,745	59.0	0.04
Statins	10,349	12.7	4,375	14.2	5,974	11.8	<0.001
Thiazide diuretics	19,215	23.6	7,785	25.2	11,430	22.7	<0.001
Treatment I = local therapy							
Breast surgery	70,120	86.2	25,122	81.2	44,998	89.2	<0.001
Radiotherapy	38,873	47.8	13,229	42.8	25,644	50.8	<0.001
Treatment II = other (neo) adjuvant systemic treatment							
Other systemic treatment with aromatase inhibitor alone	3,886	4.78	1,141	3.69	2,745	5.44	<0.001
Other systemic treatment with chemotherapy alone	40,720	50.0	18,341	59.3	22,379	44.4	<0.001
Other systemic treatment with AI + CT	16,493	20.3	3,611	11.7	12,882	25.5	<0.001

Chi-square test; ^a^Student t test.

AI, aromatase inhibitor; CAD, coronary artery disease; COPD, chronic obstructive pulmonary disease, SD, standard deviation.

**Figure 1:**
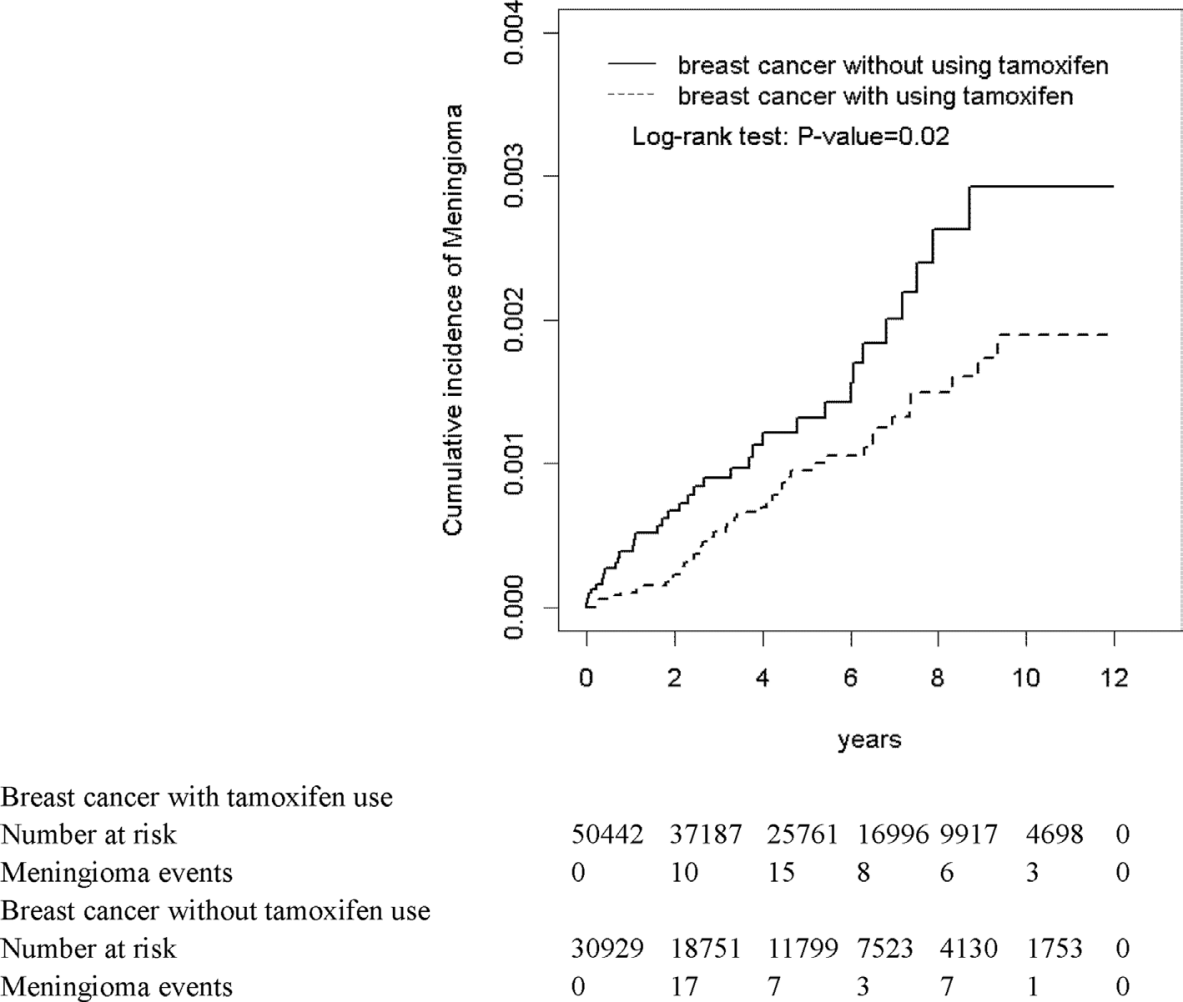
Cumulative incidence curves of meningioma for breast cancer with and without tamoxifen use.

The mean follow-up periods were 4.71 (SD = 3.30) and 3.77 (SD = 3.17) years in the tamoxifen cohort and non-tamoxifen cohort, respectively (data not shown). At the end of the 12-year follow-up period, per the Kaplan–Meier analysis, the cumulative incidence of meningioma was significantly lower in the tamoxifen cohort than in the non-tamoxifen cohort (log-rank test: *P =* 0.02) (**Figure 1**).

The overall incidence density of meningioma was lower in the tamoxifen cohort than that in the non-tamoxifen cohort (1.77 versus 3.00 per 10,000 person-years) (**Table 2**). After adjusting for age, comorbidity, steroid use, statin use, thiazide diuretics use, treatment I, and treatment II, the adjusted hazard ratio (aHR) and 95% confidence interval (CI) for meningioma was 0.64-fold (95% CI = 0.40–1.02) for the tamoxifen users as compared with non-tamoxifen users.

**Table 2:** Hazard ratios for meningioma among patients with breast cancer with and without using tamoxifen as revealed by the time-dependent regression model.

	Breast cancer without using tamoxifen	Breast cancer with using tamoxifen
No. of meningioma	35	42
Person-years	116,553	237,572
Incidence rates (per 10,000 person-years)	3.00	1.77
Crude HR (95% CI)	1.00	0.59 (0.38, 0.93)*
Adjusted HR (95% CI)^#^	1.00	0.64 (0.40, 1.02)

CI, confidence interval; HR, hazard ratio ^#^Adjusted for age, comorbidity, steroid use, statin use, thiazide diuretics use, treatment I, and treatment II. *P < 0.05.

Breast cancer patients receiving >1,500 days of tamoxifen treatment exhibited significantly decreased meningioma risk compared with breast cancer patients who had not received tamoxifen treatment (aHR = 0.42, 95% CI = 0.19–0.91) (**Table 3**). Similar results were observed among breast cancer patients with different cumulative dosages of tamoxifen use; compared with breast cancer patients who did not use tamoxifen, the patients who used >26,320 mg of tamoxifen had a significantly (aHR = 0.44; 95% CI = 0.22–0.88) lower risk of developing meningioma (**Table 4**).

**Table 3:** Adjusted hazard ratio and incidence of meningioma by duration of tamoxifen therapy in patients with breast cancer with and without tamoxifen use.

Tamoxifen exposed	*n*	Event	Person-years	IR	Adjusted HR^‡^ (95% CI)	Adjusted HR^‡^ (95% CI)
Without using tamoxifen	30,929	35	116,553	3.00	1.00	
Duration on tamoxifen						
≤365 days	17,692	11	51,514	2.14	0.69 (0.34, 1.38)	1.00
366–1,500 days	23,341	23	113,787	2.02	0.77 (0.44, 1.32)	1.00 (0.48, 2.07)
>1,500 days	9,409	8	72,271	1.11	0.42 (0.19, 0.91)*	0.51 (0.20, 1.31)

CI, confidence interval; HR, hazard ratio; IR, incidence density rates (per 10,000 person-years). ^‡^Adjusted for age, comorbidity, steroid use, statin use, and thiazide diuretics use, treatment I, and treatment II. *P < 0.05.

**Table 4:** Adjusted hazard ratio and incidence of meningioma by cumulative dosage of tamoxifen use in patients with breast cancer with and without tamoxifen use.

Tamoxifen exposed	*n*	Event	Person-years	IR	Adjusted HR^‡^ (95% CI)	Adjusted HR^‡^ (95% CI)
Without using tamoxifen	30,929	35	116,553	3.00	1.00	
Dosage of tamoxifen						
≤4280 mg	12,621	6	35,169	1.71	0.54 (0.23, 1.31)	1.00
4281–12,980 mg	12,618	13	44,173	2.94	1.07 (0.55, 2.06)	1.88 (0.71, 4.97)
12,981–26,320 mg	12,594	12	64,071	1.87	0.71 (0.36, 1.39)	1.15 (0.43, 3.09)
>26,320 mg	12,609	11	94,160	1.17	0.44 (0.22, 0.88)*	0.68 (0.24, 1.87)

CI, confidence interval; HR, hazard ratio; IR, incidence density rates (per 10,000 person-years). ^‡^Adjusted for age, aromatase inhibitor use, comorbidity, steroid use, statin use, thiazide diuretics use, treatment I, and treatment II. *P < 0.01.

**Table 5** illustrates the joint effect of aromatase inhibitor treatment and tamoxifen therapy on meningioma risk. Patients that received aromatase inhibitor treatment in addition to tamoxifen therapy did not have a significantly decreased risk of meningioma compared with patients without either treatment (aHR = 0.59, 95% CI = 0.31–1.15).

**Table 5:** Joint effects of aromatase inhibitor use and tamoxifen use on the risk of meningioma in patients with breast cancer as revealed by the time-dependent regression model.

Tamoxifen	Aromatase inhibitor	*N*	Event	Person-years	IR	Adjusted HR^‡^ (95% CI)
No	No	26,177	27	100,922	2.68	1.00
No	Yes	4,752	8	15,631	5.12	1.21 (0.54, 2.71)
Yes	No	34,815	28	156,553	1.79	0.69 (0.40, 1.18)
Yes	Yes	15,627	14	81,020	1.73	0.59 (0.31, 1.15)

CI, confidence interval; HR, hazard ratio; IR, incidence density rates (per 10,000 person-years). ^‡^Adjusted for age, comorbidity, steroid use, statin use, thiazide diuretics use, breast surgery, radiotherapy, and chemotherapy.

## Discussion

This large study used a comprehensive national database to assess the meningioma risk in patients with previous diagnoses of breast cancer with or without tamoxifen use. In our study, tamoxifen users had a non-significantly (36%) lower risk of developing meningioma than did tamoxifen non-users. However, the decreased risk became significant when patients took tamoxifen treatment for longer than 1,500 days or took a cumulative dosage larger than 26,320 mg.

The breast cancer–meningioma relationship has been investigated for decades, but the results have been discordant. Several studies have detected a positive association between these two conditions (Schoenberg et al., 1975; Helseth et al., 1989; Lieu et al., 2003; Rakha et al., 2007; Rao et al., 2009; Milano and Grossman, 2017). For example, Rao et al. found a strong association between breast cancer and meningioma, but only in women (Rao et al., 2009). Conversely, some studies have reported lower degrees of risk (Jacobs et al., 1992; Custer et al., 2002; Criscitiello et al., 2014). Rather than a causal link, this association is likely related to shared common etiological factors, for example, shared genetic predisposition (e.g., variations in DNA repair polymorphisms) and endogenous and exogenous hormones (Bethke et al., 2008; Wiemels et al., 2010).

Studies have suggested that hormonal factors may be involved in regulating the growth of meningioma (Hsu et al., 1997; Wahab and Al-Azzawi, 2003; Lee et al., 2006; Korhonen et al., 2010; Cea-Soriano et al., 2012; Ji et al., 2016). Researchers have indicated that hormone replacement therapy (especially estrogen-only) increases the risk of meningioma (Anderson et al., 2013; Benson et al., 2015). Previous studies have found that meningiomas tend to express progesterone receptors more frequently (32.1%–86.3%) than estrogen receptors (7.1%–50%) (Lieu et al., 2003; Wahab and Al-Azzawi, 2003; Korhonen et al., 2010), suggesting that progesterone and estrogen may influence tumor growth. Progesterone and estrogen antagonists may therefore inhibit tumor growth. An early study has found that selected meningiomas are subject to hormonal influence *in vitro*, and the inhibition of meningioma growth *in vitro* was observed by the antiprogesterone (Olson et al., 1986). Antiprogesteronal therapy and antiestrogenic therapy have been proposed for managing meningiomas (Markwalder et al., 1985; Goodwin et al., 1993; Grunberg, 1994; Ji et al., 2015); however, no definite role has been confirmed. Generally, tamoxifen binds to estrogen receptors and acts as partial antagonist or agonist depending on the type of target tissue (Yeh et al., 2014). Two studies have focused on the treatment effect of tamoxifen in refractory, recurrent, or unresectable meningiomas, but neither indicated a favorable response of meningiomas to antiestrogenic therapy (Goodwin et al., 1993; Grunberg, 1994). Regardless of the null therapeutic effect, investigators have been interested in the prophylactic role of tamoxifen in meningioma among breast cancer patients. For example, Ji et al. evaluated the association of tamoxifen with meningioma in the Swedish population and reported that women with breast cancer who did not use tamoxifen had increased meningioma incidence, whereas in breast cancer patients treated with tamoxifen, the incidence was nearly the same as that of the general population, which suggests that tamoxifen likely plays in preventing meningioma development (Ji et al., 2016). Although the current study could not conclusively establish that for breast patients, tamoxifen treatment significantly decreases the risk of subsequent meningioma compared with non-tamoxifen treatment, the results were borderline significant, with a direction (protective role) consistent with their findings. The potential mechanisms account for this discrepancy of treatment and inverse association of tamoxifen in meningioma is still undetermined. We have to acknowledge that the information regarding mechanism of action and pharmacokinetic profiles of tamoxifen in meningothelial cells is scarce. Furthermore, the possible molecular mechanism(s) in the link between tamoxifen and meningioma is undetermined yet.

The NHI patient records provide a unique resource for a nationwide investigation of meningioma incidence among breast cancer patients. Other available and useful information was also retrieved for this study. The etiology of meningioma remains unclear, and ionizing radiation exposure and chemotherapy agents may be risk factors for meningioma development (Braun et al., 2004; Umansky et al., 2008; Wiemels et al., 2010). The basic treatment information is available in the NHIRD, and we have adjusted these two factors in our analyses.

Tamoxifen in Taiwan is normally prescribed in breast cancer patients with a daily dose of 20 mg for a 5-year continuous treatment. Stratified analysis by treatment duration and cumulative dosage revealed that the inverse association of tamoxifen in meningioma among breast cancer patients was limited to those patients with long-term use (>1,500 days) and dosage larger than 26,320 mg. The compliance of patients to tamoxifen may be related to the long-term use and total dosage, and it may imply that good compliance of patients to tamoxifen can reduce the risk of meningioma. Earlier studies from NHIRD evaluating the compliance of other drugs also supported this inference (Li and Huang, 2015; Tung et al., 2017). We speculate that meningiomas are dependent on estrogen receptor (ER) as one of the reasons for the positive correlation between long-term tamoxifen use and reduced meningioma incidence. Presently, prescriptions that last longer than 5 years are uncommon as an adjuvant therapy for breast cancer. However, extending the duration of adjuvant treatment to 10 years has been previously reported to have a reduced risk of late breast cancer recurrence, thus improving survival (Davies et al., 2013). **Table 4** also displays a possible biphasic dose–response curve; however, the possible reasons accounting for this phenomenon are unclear. A previous paper has assessed hormone replacement therapy and the risk of meningioma and found that the risk of meningioma increases with duration of use of combined estrogen–progestagen within 1 year and ≥10 years, which also revealed a biphasic duration–response relationship (Anderson et al., 2013). We acknowledge that the underlying mechanisms may be worth exploring in other studies. No significant joint effect was observed for treatment with tamoxifen and aromatase inhibitors. Similarly, no evidence was noted regarding whether the use of aromatase inhibitors affects the association between tamoxifen and meningioma. In addition, aromatase inhibitors without a similar association may be related to different mechanisms of anti-estrogen effect. Tamoxifen is known to antagonize activation function (AF)-2 in estrogen receptor (ER) but selectively activate estrogen receptor (ER)-AF1 and the AF1-mediated specific target genes, which are associated either with meningioma development or prevention. On the other hand, aromatase inhibitors work by inhibiting the action of the enzyme aromatase, which converts androgens into estrogens by aromatization. A previous study has found that there is a significantly increased risk of meningioma among men users of androgen analogues (Cea-Soriano et al., 2012), and we speculate that this may partially account for the null protective effect of aromatase inhibitors in the development of meningioma.

To the best of our knowledge, this research is the first study with nationwide coverage in an Asian country to focus on consequent meningioma incidence among women with breast cancer taking tamoxifen. A major strength of the present study is that it used a nationwide population-based database for comprehensive analyses, which increases the statistical power of the study and generalizability. However, the database has several intrinsic limitations. First, the NHIRD does not contain detailed histology and phenotype information regarding breast cancer and meningioma; thus, we cannot control for breast cancer subtype when comparing the differences between the use of tamoxifen. We cannot evaluate the exact estrogen receptor and progesterone receptor statuses to further correlate the hormone effects on the possible relationship between tamoxifen and meningioma, either. Furthermore, grade of meningiomas cannot be differentiated from NHIRD, and it hinders us to assess if tamoxifen use or not has different effects on different grades of meningioma. Milano et al. found that women with both breast cancer and meningioma tended to have more advanced breast cancers and smaller-sized meningiomas compared with women having only one of these diagnoses (Milano and Grossman, 2017); unfortunately, the current study cannot justify it. Second, precise brain MRI and CT information is unavailable in the NHIRD, and the resulting potential surveillance bias precludes a fair comparison of the risk of meningioma in breast cancer patients with that in the normal population. This might not be a big issue to be concerned since the central neural system (CNS) is not routinely screened in breast cancer patients, unless for patients with symptoms. On the other hand, meningioma is not systematically screened by brain MRI or CT, and the tumor may be found incidentally on an MRI or CT scan performed for other reasons. Third, information relevant to the lifestyle and behavior of the patients is scarce in the NHIRD, which made it difficult to adjust for the health behavior–related factors, such as smoking and unhealthy diets, which are well documented to increase the risk of certain cancers (Schwarz et al., 2007); moreover, the association of cigarette smoking and meningioma case status varies significantly by gender (Claus et al., 2012). Fourth, we realized that the comparator groups are not balanced in some factors as shown in **Table 1**; thus, we did the adjustment for these factors. However, residual confounding may still exist even after adjustment. Fifth, body mass index information is not recorded in the NHIRD, so we cannot adjust this factor in the analyses, either. Finally, tamoxifen user and non-user groups do not share cancer phenotypes. In general, patients who were prescribed tamoxifen were hormone receptor-positive, while those who were not prescribed tamoxifen were hormone receptor-negative. The former patients were known to have significantly better survival than the latter (Cianfrocca and Goldstein, 2004; Ren et al., 2014). The statistical data provided in this study could be misinterpreted since the comparison analysis in this specific setting can be compromised by other phenotypic variables. In addition, meningioma is typically a slow-growing tumor, and many cases never produce any symptoms. The risk of meningioma is higher in tamoxifen non-users partially because they were more likely to receive brain MRI or CT, for which they were easier to metastasize to brain compared with tamoxifen users. However, the current database cannot adjust for such confounding factor. Although some intrinsic limitations were imposed by the database, the data used in this study regarding the diagnoses of breast cancer and meningioma, the use of tamoxifen, as well as other treatments of breast cancer are highly trustworthy.

In summary, although primary hypothesis testing that tamoxifen use can decrease the risk of meningioma was not statistically significant, a trend of decreased risk of meningioma development among Taiwanese breast cancer survivors treated with tamoxifen was observed, especially for those with a long duration or a high dosage of tamoxifen therapy. The plausible mechanisms for the potential protective involvement of tamoxifen treatment in the meningioma development remain to be defined clearly. Although this could be explained partially by the hormone factor, additional comprehensive studies are warranted, and confirmatory evidence would be required before appropriate recommendations can be made.

## Ethics Statement

The NHIRD encrypts patient personal information to protect privacy and provides researchers with anonymous identification numbers associated with relevant claims information, including sex, date of birth, medical services received, and prescriptions. Therefore, patient consent is not required to access the NHIRD. This study was approved to fulfill the condition for exemption by the Institutional Review Board (IRB) of China Medical University (CMUH104-REC2-115-CR3). The IRB also specifically waived the consent requirement.

## Author Contributions

Conception and design: LS and CK; administrative support: CK; collection and assembly of data: all authors; data analysis and interpretation: all authors; manuscript writing: all authors; final approval of manuscript: all authors.

## Funding

This study is supported in part by Taiwan Ministry of Health and Welfare Clinical Trial Center (MOHW108-TDU-B-212-133004), China Medical University Hospital (CMU106-ASIA-12, DMR-108-207), Academia Sinica Stroke Biosignature Project (BM10701010021), MOST Clinical Trial Consortium for Stroke (MOST 108-2321-B-039-003-), Tseng-Lien Lin Foundation, Taichung, Taiwan, and Katsuzo and Kiyo Aoshima Memorial Funds, Japan.

## Conflict of Interest Statement

The authors declare that the research was conducted in the absence of any commercial or financial relationships that could be construed as a potential conflict of interest.

## Abbreviations

HR, hazard ratio; CI, confidence interval; NHIRD, National Health Insurance Research Database; ICD-9-CM, International Classification of Diseases, Ninth Revision, Clinical Modification; COPD, chronic obstructive pulmonary disease; CAD, coronary artery disease
